# Time to Reset: The Interplay Between Circadian Rhythms and Redox Homeostasis in Skeletal Muscle Ageing and Systemic Health

**DOI:** 10.3390/antiox14091132

**Published:** 2025-09-18

**Authors:** Elizabeth Sutton, Vanja Pekovic-Vaughan

**Affiliations:** Institute of Life Course and Medical Sciences, Department of Musculoskeletal & Ageing Science, University of Liverpool, Liverpool L7 8TX, UK; ems33@liverpool.ac.uk

**Keywords:** skeletal muscle, ageing, circadian rhythms, NRF2, redox homeostasis, oxidative stress, chronotherapy, chronotype, exercise, sarcopenia, frailty, myokines

## Abstract

Skeletal muscle plays vital roles in locomotion, metabolic regulation and endocrine signalling. Critically, it undergoes structural and functional decline with age, leading to a progressive loss of muscle mass and strength (sarcopenia) and contributing to a systemic loss of tissue resilience to stressors of multiple tissue systems (frailty). Emerging evidence implicates misalignments in both the circadian molecular clock and redox homeostasis as major drivers of age-related skeletal muscle deterioration. The circadian molecular clock, through core clock components such as BMAL1 and CLOCK, orchestrates rhythmic gene, protein and myokine expression impacting diurnal regulation of skeletal muscle structure and metabolism, mitochondrial function, antioxidant defence, extracellular matrix organisation and systemic inter-tissue communication. In parallel, the master redox regulator, NRF2, maintains cellular antioxidant defence, tissue stress resistance and mitochondrial health. Disruption of either system impairs skeletal muscle contractility, metabolism, and regenerative capacity as well as systemic homeostasis. Notably, NRF2-mediated redox signalling is clock-regulated and, in turn, affects circadian clock regulation. Both systems are responsive to external cues such as exercise and hormones, yet studies do not consistently include circadian timing or biological sex as key methodological variables. Given that circadian regulation shifts with age and differs between sexes, aligning exercise interventions with one’s own chronotype may enhance health benefits, reduce adverse side effects, and overcome anabolic resistance with ageing. This review highlights the essential interplay between circadian and redox systems in skeletal muscle homeostasis and systemic health and argues for incorporating personalised chrono-redox approaches and sex-specific considerations into future experimental research and clinical studies, aiming to improve functional outcomes in age-related sarcopenia and broader age-related metabolic and musculoskeletal conditions.

## 1. Introduction to Skeletal Muscle: From Structural to Metabolic and Endocrine Functions

The human body comprises >600 different skeletal muscles, accounting for ~40–60% of total body mass. Skeletal muscles contain ~50–75% of all proteins within the body and are responsible for ~30–50% of all protein turnover [1]. Certain properties of skeletal muscles are determined from an early developmental stage, such as anatomical structure, shape, spatial organisation and orientation, along with connections to correct tendons and bones [2]. Yet, skeletal muscle is one of the most dynamic and plastic tissues within the body, with skeletal muscle mass, force and fatigue resistance being adaptive to physiological or pathological conditions. For example, exercise training regimens can lead to increased skeletal muscle mass in young adults, alongside improved force and resistance to fatigue, whilst in disease conditions or with ageing, skeletal muscles lose mass and/or strength leading to sarcopenia and associated frailty [2].

**Figure 1:**
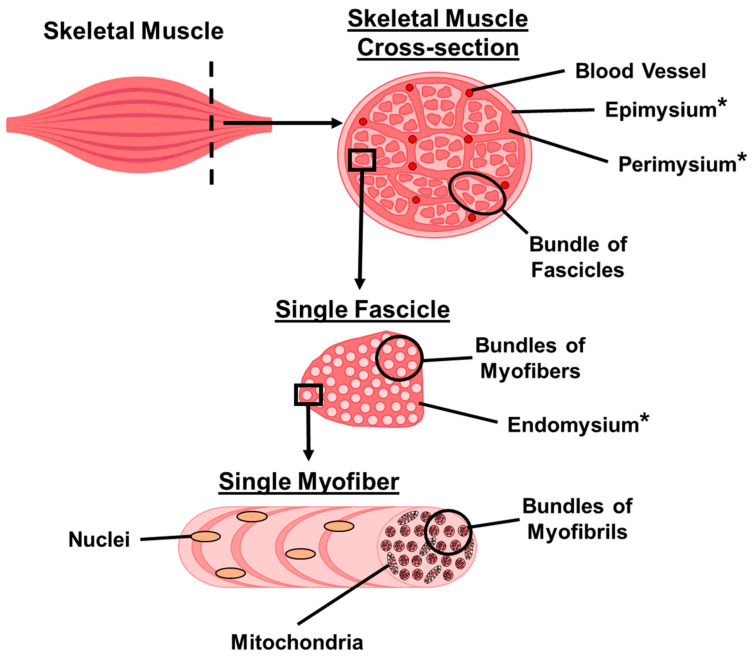
**Schematic depicting a highly organised structure of skeletal muscle.** Skeletal muscle is one of the most abundant tissues in the body, possessing structural, metabolic and endocrine functions. Skeletal muscle is a highly organised tissue composed of individual myofibrils. The myofibrils bundle into a single myofibre, which contains many nuclei and mitochondria. The myofibres are bundled to create a fascicle. The bundles of myofibres are supported by a layer of extracellular matrix (ECM) called the endomysium. The fascicles bundle to form the skeletal muscle. The fascicles are supported by an ECM layer called the perimysium, and they are highly oxygenated through a blood supply. An additional layer of ECM called the epimysium surrounds the whole skeletal muscle. The epimysium mainly contains types I, III collagens and fibronectin; the perimysium contains collagen types I, III, V and VI, proteoglycans and fibronectin, while the endomysium contains types I, III, and V collagens, laminins and fibronectin. * Supportive ECM.

The highly organised architecture of skeletal muscle is well-characterised. Briefly, myofibrils form the basis of the skeletal muscle structure, composed of thick myosin filaments and thin filaments of actin, troponin and tropomyosin. The myofibrils are bundled to form myofibres, the myofibres themselves are bundled to create the fascicles, and the bundles of the fascicles form the skeletal muscle [3,4] (see Figure 1). A complex network of supportive extracellular matrix (ECM) consisting of collagens, glycoproteins, proteoglycans and elastin [5] surrounds the myofibres and the fascicles, providing mechanical support [6]. Skeletal muscle-specific stem cells known as satellite cells reside within the ECM, where they exert key functions in myogenesis and muscle morphogenesis, thereby playing an important role in the growth and repair of skeletal muscle in response to injury, disease or sedentary behaviour [7].

Extracellular matrix (ECM) is the non-cellular structural scaffold which surrounds all tissues and organs, with >1000 characterised proteins, called the matrisome. The ECM is essential for tissue growth and remodelling as well as stabilising tissue structures and providing elasticity [8]. The ECM is particularly vital in supporting the function of skeletal muscle, providing important cues for transmission of force and regulating skeletal muscle homeostasis through myogenesis, repair and degradation [9]. The thickest and strongest of the structures of ECM, the epimysium, covers the whole skeletal muscle (see Figure 1). The tendon that joins the skeletal muscles to the bones continues as the epimysium, which contains the main blood vessels and nerves that nourish the skeletal muscles. There are many myofibres that surround the ECM layer called the perimysium to form fascicles. The perimysium contains larger blood vessels and nerves and transmits the force to tendons. Each muscle fibre is encircled by a thin and delicate component of the ECM, called the endomysium. Small arteries and neurons are found in the endomysium, which plays a significant role in myogenesis, regeneration and preserves the integrity of the skeletal muscle [10]

Skeletal muscle is involved in a wide array of physiological functions, which can be broadly split into three groups: mechanical, metabolic and endocrine. The mechanical functions of skeletal muscle enable the maintenance of posture, generation of movement and maintenance/improvement of health through the conversion of chemical into mechanical energy [1]. In terms of metabolism, skeletal muscle is responsible for the uptake of ~80% glucose following a meal [11], and is involved in the regulation of energy metabolism and heat production for thermoregulation through the mitochondrial consumption of oxygen and fuel during daily activity. Skeletal muscle also acts as a metabolic reserve of amino acids following nutritional intake. In addition, during times of poor nutritional intake, it helps provide those amino acids to tissues that need maintenance of proteostasis for survival, such as the brain and heart, though this can only occur if skeletal muscle mass is adequate [12].

Skeletal muscle has endocrine functions that have crucial roles in inter-tissue communication and systemic health effects. This was first proposed by Goldstein ~60 years ago, who viewed skeletal muscle as an endocrine-like organ, releasing an unidentified humoral factor involved in regulating blood glucose in response to exercise [13]. Later in the early 2000s, Pedersen and colleagues identified and demonstrated in a series of studies that interleukin-6 (IL-6), irisin and brain-derived neurotrophic factor (BDNF), produced by skeletal muscles during contraction, play an important role in the beneficial health effects of exercise by mobilising extracellular substrates and/or supporting substrate delivery during exercise [14,15,16]. Further research established that skeletal muscle produces numerous cytokines, peptides and metabolites, termed myokines [17]. Hartwig and colleagues catalogued the secretome of human primary muscle cells, identifying 548 non-redundant proteins in conditioned media, with 305 proteins classified as potential myokines [18]. These myokines exhibit autocrine, paracrine and endocrine effects on various cells and tissues, placing skeletal muscle as an endocrine organ [19]. 

Exercise-induced myokine release, termed exerkines, contribute to the broad beneficial effects of physical activity on overall health. They control adaptive processes in skeletal muscle by acting as regulators of hypertrophy, angiogenesis, inflammation and ECM remodelling, and in doing so, mediate communication between skeletal muscle and other organs, including the brain, adipose tissue (AT), pancreas, liver, intestines and other musculoskeletal tissues [20]. The interaction between skeletal muscle and adipose tissue, both white (WAT) and brown (BAT), has been of great metabolic interest, with WAT being a key energy storage depot, releasing hormones and cytokines to regulate whole body metabolism, and BAT being necessary for thermogenesis. Both skeletal muscle and AT are affected by exercise and help the body to adapt to metabolic challenges. The tissue crosstalk, through the release of myokines, mediates inter-tissue communication to improve total metabolic health [21]. Some examples of myokines include IL-6, which leads to increases in glucose uptake and β-oxidation within the skeletal muscle itself, but also promotes lipolysis in WAT and glucagon-like peptide-1 (GLP1) secretion in the pancreas and gut that help insulin release to regulate blood sugar levels. Other examples include IL-15, which induces hypertrophy in skeletal muscle, as well as increases thermogenesis and β-oxidation in BAT, whilst BDNF increases β-oxidation in skeletal muscle and promotes learning and memory in the brain [20].

Skeletal muscle extracellular vesicles (EVs) have emerged as another crosstalk mechanism through which the beneficial effects of exercise are transmitted to other tissues. EVs are membrane-bound vesicles with the capacity to transfer functional biomolecules such as lipids, proteins, nucleic acids and sugars. EVs are currently divided into three subtypes: exosomes (50–150 nm in diameter), microvesicles (100–1000 nm) and apoptotic bodies (50–5000 nm) [22]. Mitochondrial DNA was found to be taken up into exosomes and released into extracellular medium of C2C12 cell cultures [23]. Several miRNAs are highly abundant in skeletal muscle including *miR-1, miR-133a, miR-133b* and *miR-206*. These account for ~25% of miRNA expression in human skeletal muscle, contributing to myoblast differentiation via an autocrine route, and become excreted from skeletal muscle cells via exosomes [24]. A further study by Guescini and colleagues reported that circulation of *miR-133b* and *miR181a-5p* in exosomes was increased following acute exercise, with a positive correlation between aerobic fitness and circulating skeletal muscle-abundant miRNAs [25]. It has been suggested that miRNA-containing exosomes excreted from the skeletal muscle mediate the stress response of cells by regulating gene expression to restore homeostasis. Resistance training has been shown to reduce levels of *miR-1* and *miR-133a*, targeting the insulin-like growth factor 1 (IGF1) pathways and inducing hypertrophy, whilst endurance training increased *miR-1* and *miR-181* to induce mitochondrial biogenesis via the peroxisome proliferator-activated receptor gamma coactivator 1-α (PGC-1α) pathway [26].

## 2. Age-Associated Sarcopenia and the Role of Redox Homeostasis

Sarcopenia is an age-related condition characterised by the loss of skeletal muscle mass, quality and strength, the rate of which is dependent on activity levels, co-morbidities, nutritional status and circadian regulatory factors. Given its structural, metabolic and endocrine roles, skeletal muscle loss can have serious functional and metabolic consequences on the body, including increased risk of frailty that is characterised by reduced resilience to stressors, physical immobility and a functional decline in several organ systems such as cardiovascular, cognitive and metabolic [27]. As skeletal muscle undergoes pathophysiological changes with ageing, the functions of its major components such as myofibres, ECM, satellite cells and mitochondria become adversely affected.

Type II myofibres undergo a significant atrophy with ageing and an irreversible decline in number due to loss of innervation [28]. Several studies have determined that in the elderly, type II myofibres become more deformed from their typical tessellations of pentagons or hexagons seen in younger adults, though type I myofibres appear to be less affected by ageing [29,30]. The reduction in type II myofibre number directly results in a loss of myosin heavy chain (MHC) isoforms IIa and IIx, which are associated with strength and speed of muscle contraction. Contractile properties of older adults are therefore weaker, slower and less powerful than that of younger adults [31]. The loss of myosin impedes the formation of actin–myosin cross-bridges required for contractility, further impairing skeletal muscle activity [32]. Loss of muscle fibres is associated with the loss of motor units, leading to myofibre denervation, and increased susceptibility to atrophy [33].

Protein homeostasis of the ECM is severely impacted with age resulting in loss of new ECM protein synthesis, reduced turnover of old ECM and increased matrix metalloproteinase degradation activity [34]. During ageing, collagens, mainly type I and III, undergo glycation and form harder-to-cleave crosslinks. Furthermore, collagens can no longer align in linear conformations, and deteriorate into erratically grouped fibres, thus stiffening the matrix and inducing the build-up of fibrotic connective tissue within the skeletal muscle [34,35,36]. Another consequence of skeletal muscle ageing is the diminishing function of the satellite cell (SC) niche within the ECM. Upon skeletal muscle injury in young adults, the satellite cells proliferate and differentiate into new muscle fibres. However, with age, satellite cells are driven towards a fibro-adipogenic fate leading to fibrosis and impaired recovery from injury [37]. In addition, the increased deposition of basal lamina proteins with skeletal muscle ageing can drive SCs out of their niches, affecting the regulation of their divisions [10].

Furthermore, in line with the mitochondrial theory of ageing, mitochondrial mass, oxygen consumption and ATP synthesis become impaired with ageing with a ~40–50% decrease in oxidative phosphorylation, resulting in a major decline in energy homeostasis [38]. Reactive oxygen and nitrogen species (RO/NS) are produced in cells predominately as a by-product of mitochondrial oxidative phosphorylation. Build-up of RO/NS can be detrimental to nucleic acids, proteins and organelles, thus cells and tissues have evolved a protective network of detoxifying enzymes and non-enzymatic antioxidants to prevent excessive RO/NS accumulation [39]. This cellular antioxidant defence system is controlled by the master antioxidant transcription factor NRF2 [40].

**Figure 2:**
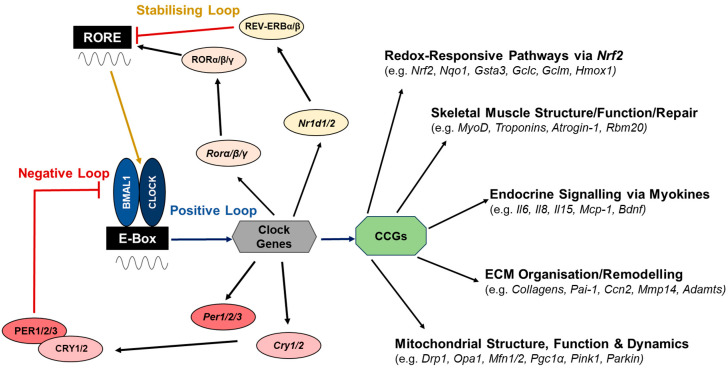
**Schematic depicting the molecular circadian clock TTFL mechanism and downstream CCGs and molecular pathways under circadian control.** BMAL1 and CLOCK within the positive arm of the molecular clock heterodimerise to activate rhythmic transcription of the negative and stabilising loop target genes as well as numerous clock-controlled genes (CCGs) by binding to E-box elements in the gene promoters. PER1/2/3 and CRY1/2, the negative loop components, inhibit BMAL/CLOCK activity to repress their own expression and that of CCGs. The stabilising loop components activated by the BMAL/CLOCK heterodimer include RORα/β/γ and REV-REBα/β, which regulate rhythmic expression of core clock genes such as *Bmal1* and *Clock* in a positive or negative manner, respectively, via binding to ROREs in the gene promoters. Examples of downstream CCGs within various molecular pathways under circadian control include the following: redox-responsive pathways via *Nrf2*, pathways regulating muscle structure, function and repair, endocrine signalling via myokines and pathways regulating ECM homeostasis and mitochondrial structure, function and dynamics.

NRF2 belongs to the Cap ‘n’ Collar (CNC) basic region-leucine zipper (bZIP) family [41] and its main endogenous regulator is KEAP1 (Kelch-like erythroid cell-derived protein with CNC homology-associated protein 1), an E3 ubiquitin ligase substrate adaptor. Under low oxidative stress, KEAP1 sequesters NRF2 in the cytoplasm, enabling the binding of E3 ligase complex CULLIN3, which polyubiquitinylates NRF2 leading to its proteasomal degradation [42]. Upon cellular oxidative stress, cysteines on KEAP1 become oxidised, so that NRF2 can no longer be sequestered for degradation, thus allowing de novo synthesised NRF2 to accumulate in the cytoplasm and translocate to the nucleus. In the nucleus, NRF2 heterodimerises with small musculoaponeurotic fibrosarcoma (sMAF) proteins, binds to antioxidant response elements (AREs) within the promoter regions of cytoprotective genes encoding proteins such as phase 2 and antioxidant enzymes, and stimulates their timely transcription. Some of the NRF2-dependent antioxidant enzymes include Quinone oxidoreductase 1 (*Nqo1*)*,* Heme oxygenase-1 (*Hmox-1*), Peroxiredoxin-1 (*Prdx-1*) and glutathione enzymes such as Glutamate–cysteine ligase catalytic subunit (*Gclc*), Glutamate–cysteine ligase modifier subunit (*Gclm*) and Glutathione S-transferases (*Gsts*) [43] (see Figure 2). Other antioxidant proteins work independently of NRF2, such as mitochondrial coenzyme Q10, a key cofactor of the electron transport chain which scavenges RO/NS [44] and vitamin E, which down-regulates the generation of RO/NS and superoxide within mitochondria, thereby attenuating oxidative damage [45].

With age, there is a shift in redox homeostasis to a more oxidative environment, with the accumulation of RO/NS via impaired mitochondrial function and/or defective RO/NS elimination processes, and this build-up of oxidative stress with age has been implicated in the development of sarcopenia [46]. In addition, nuclear accumulation of NRF2 is reduced with ageing, leading to impaired cytoprotective responses to oxidative stress and reduced elimination of RO/NS [47]. In older mice lacking *Nrf2*, markers of oxidative stress burden were robustly increased, alongside decreased expression of antioxidant genes, rate and efficiency of respiration, leading to the higher production of RO/NS per O_2_ consumed. Loss of *Nrf2* with age exacerbated age-associated mitochondrial oxidative stress [48], impaired skeletal muscle mitochondrial biogenesis and dynamics and worsened both sarcopenia and frailty [49]. Loss of *Nrf2* in mice also triggered ubiquitination and pro-apoptotic signals in the skeletal muscle, leading to protein degradation, cell death and skeletal muscle deterioration with age [50,51]. Thus, *Nrf2* plays a protective role against early development of sarcopenia, as older *Nrf2* KO mice had increased RO/NS production and reduced O_2_ consumption by the mitochondria, along with reduced skeletal muscle mass and diminished generation of contractile force [52] . In line with this, sedentary older wild type mice also showed an impaired *Nrf2-Keap1* signalling axis, which promoted oxidative damage to skeletal muscle and impaired redox homeostasis [53].

## 3. Introduction to the Circadian Clock Timing System

**Figure 3:**
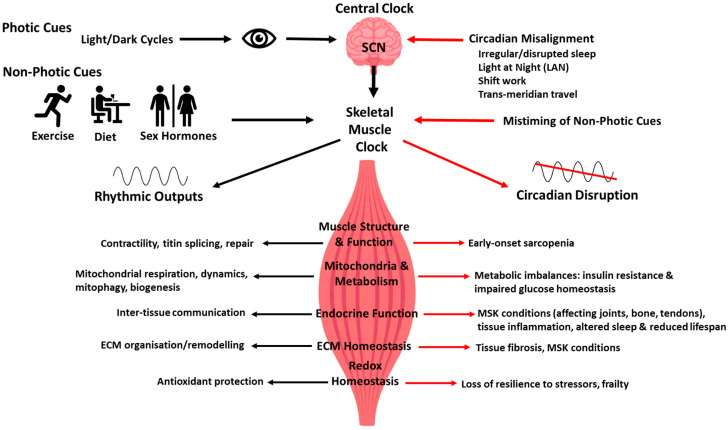
**Schematic depicting the roles of mammalian skeletal muscle clock in regulating tissue-specific rhythmic outputs and systemic health and the consequences of circadian disruption**. Photic cues (e.g., light/dark cycles) entrain the mammalian SCN clock, a central clock within the brain’s anterior hypothalamus. SCN relays photic information through the retina to the peripheral clocks in tissues and cells around the body, including skeletal muscle, to coordinate their synchronisation to the external environment. Non-photic cues can also synchronise the peripheral clocks independently of SCN. Skeletal muscle clock leads to several key rhythmic outputs encompassing its structural, metabolic and endocrine roles. Circadian misalignments in light/dark cycles or mistiming of non-photic cues lead to functional consequences to skeletal muscle homeostasis (e.g., early-onset sarcopenia and frailty) as well as systemic health effects, such as metabolic imbalances, MSK conditions affecting joints, bone and tendons, tissue fibrosis and inflammation, altered sleep and reduced lifespan.

Organisms from across the three domains of life, bacteria, archaea and eukaryotes, all display time-of-day dependent changes to their physiology, behaviour and metabolism, which ensures their ability to adapt to daily environmental changes [54]. This evolutionarily conserved, endogenous ~24 h time-keeping system is termed the circadian clock (from the Latin ‘circa diem’ meaning ‘around a day’), which is responsible for the generation of biological oscillations in physiological and metabolic processes called the ‘circadian rhythms’. Some of the daily physiological processes exhibiting circadian rhythms include the sleep/wake cycles, thermoregulation, energy homeostasis, metabolic homeostasis, hormone secretion and locomotor activity [55]. In mammals, according to the traditional model, circadian clock regulation is hierarchical with the master or central clock located in the suprachiasmatic nuclei (SCN) of the brain’s anterior hypothalamus receiving the main entrainment signal from light, and coordinating the synchronicity of peripheral clocks found in various tissues and cells around the body, such as skeletal muscle, lungs and liver. The light-dependent time cues from the SCN are relayed via the retina through the neuronal and endocrine pathways to the peripheral clocks (SCN-dependent synchronisation) [56]. Recent research suggests a bidirectional circadian network system, whereby peripheral clocks can also maintain their rhythms independently of the SCN through responding to external non-photic cues such as cycles of feeding/fasting, activity/rest and drug administration (SCN-independent synchronisation), with feedback signals from these tissues acting upon the master clock [57]. Additional features of the circadian timing system include inter-tissue communication via hormones, myokines and metabolites, which integrate the whole circadian system and regulate the body’s response to daily cues; see Figure 3 [58,59,60]. These external cues produce minor but biologically predictable circadian shifts, many of which optimise the body’s response to a challenge [61]. Circadian misalignment, a mismatch between the internal circadian clock and the daily external cues (e.g., light/dark cycles, activity/rest or feeding/fasting cycles), can be caused by factors such as irregular or disrupted sleep, light pollution (e.g., light at night (LAN) exposure), shift work or frequent transmeridian travel, and has been associated with many diseases including metabolic disorders, cardiovascular diseases, musculoskeletal conditions, immune system impairments, neurodegenerative diseases and cancer. 

The SCN, present in the anterior region of the brain’s hypothalamus, is a bilateral structure, composed of two nuclei comprising 10,000 neurons each [62]. The SCN can be sub-divided into the dorsal ‘shell’ and ventral ‘core’ regions. The ‘shell’ contains cells expressing arginine vasopressin (AVP), whilst the ‘core’ contains gastrin-releasing peptide (GRP) and vasoactive intestinal peptide (VIP) [63]. Entrainment of mammals to the environmental ~24 h light/dark cycle is regulated by the retino-hypothalamic tract (RHT) via direct input from photoreceptive retinal ganglion cells, which depolarises the retinorecipient neurons in the ventral core of the SCN [64]. γ-aminobutyric acid (GABA) acts as the primary inhibitory neurotransmitter in the brain [65]. GABA propagates the signal received by the SCN core to the dorsal SCN shell [64]. The SCN clock projects to other regions of the brain, which are responsible for regulating brain-specific behavioural, autonomic and neuroendocrine circadian rhythms, such as cognition, mood and brain homeostasis. The SCN clock utilises the autonomic, neuroendocrine and behavioural pathways, via hormones and temperature, as systemic cues to synchronise clocks of peripheral tissues. The peripheral clocks regulate tissue-specific physiological rhythms, whose correct functioning is required for optimal health, including blood pressure, glucose metabolism and skeletal muscle force and function [64,66].

## 4. The Molecular Network Regulating the Clock Circuitry

The mammalian circadian rhythms are governed by the molecular clock mechanism that relies on the interlocked network of auto-regulatory transcriptional/translational feedback loops (TTFLs), which leads to rhythmic expression of genes, proteins and metabolites. Temporal homeostasis is maintained as the molecular clock responds to metabolic and environmental stimuli [67]. The molecular clock comprises positive, negative and stabilising feedback loops; see Figure 2. The positive arm is formed by the basic helix-loop-helix-PER-ARNT-SIM (bHLH-PAS) transcription factors CLOCK (Circadian locomotor output control kaput) and BMAL1 (Brain muscle arnt-like 1) [68]. CLOCK and BMAL1 heterodimerise to activate the transcription of core clock genes, as such the negative loop genes *Per1/2/3 and Cry1/2*, by binding to E-box elements in their gene promotor regions. The PER (Period) and CRY (Cryptochrome) proteins accumulate in the cytoplasm before forming multimers, translocate back to the nucleus and inhibit CLOCK:BMAL1 transcriptional activity, thereby leading to their own repression [54]. The PER and CRY proteins are regulated by phosphorylation through casein kinase I δ/ε and AMP-regulated protein kinase (AMPK), respectively, which leads to their ubiquitination and subsequent degradation, which plays a crucial role in regulating the period length of the circadian cycle [69]. As PER and CRY levels decrease, this enables the CLOCK:BMAL1 heterodimer to re-initiate transcriptional activity. The feedback mechanism between the positive and negative arms of the molecular clock takes ~24 hours to complete and represents the driving force behind circadian rhythm generation [68].

The CLOCK:BMAL1 heterodimer also initiates the E-box mediated transcription of the stabilising loop components which act to fine-tune the molecular clock. The stabilising arm consists of the retinoic acid-related orphan receptors (RORs) encoded by *Rorα/β/γ* and nuclear receptor subfamily 1, group D (REV-ERBs) encoded by *Nr1d1/2*. RORs activate *Bmal1* and *Clock* transcription via ROR-response elements (RORE) in their gene promotors, whilst REV-ERBs repress their transcription [67,68]. In addition, numerous clock-controlledgenes (CCGs) are transcriptionally activated by the CLOCK:BMAL1 heterodimer. A total of ~43% of the mammalian genome is expressed in a circadian manner, and across any two tissues <10% of genes are shared, suggesting a high level of tissue specificity [70]. The core molecular clock regulates thousands of CCGs in a cell type- and tissue-specific manner that are outside of the molecular core clock mechanism, and the protein products of some of these targets can often feedback onto the core clock mechanism. CCGs rhythmically oscillate in their expression, and many encode for transcription factors or proteins involved in rate-limiting steps in metabolic pathways, thereby producing rhythmic changes in tissue-specific physiology [71]. Figure 2 details the molecular clock mechanism underlying the circadian rhythm generation and examples of downstream CCGs within various molecular pathways under circadian control discussed in sections below.

## 5. Skeletal Muscle Clock: From Experimental Models to Human Studies

Skeletal muscle has been known to be under circadian regulation for well over two decades. Functions regulated by the skeletal muscle clock range from mitochondrial biogenesis and oxidative metabolism, skeletal muscle force and function [66], titin splicing [72], proteostasis [73] to satellite cell-mediated muscle regeneration and repair [74]. More recent work has shown that the skeletal muscle clock also regulates systemic health effects ranging from musculoskeletal homeostasis of bones, tendons and joints [75] to glucose homeostasis [76] and total sleep amount [77]; see Figure 3.

One of the first reports showing skeletal muscle as a peripheral tissue clock was in 1998, when Zylka and colleagues discovered the circadian expression of the equine *Per* genes [78]. Miller and colleagues were the first to report the presence of the circadian transcriptome in mouse skeletal muscle [79]. Further investigations through global transcriptomic profiling identified >800 genes which oscillate in *Gastrocnemius* mouse skeletal muscle [76,80]. A large number of discovered skeletal muscle CCGs are involved in physiological processes specific to the skeletal muscle, such as Myoblast determination protein (*MyoD)*, Troponins and *Atrogin-1* [81]; see Figure 2. With the continuing development of bioinformatic analysis tools, Harfmann and colleagues were able to expand the number of genes expressed in a circadian manner within skeletal muscle to >2300 [82]. Further transcriptomic data for mouse *Tibialis anterior* (TA) and *Soleus* muscles identified a clock-controlled output of ~1000–1500 genes, and these two muscles only shared ~110 CCGs [83]. Overlapping genes included all core clock genes as well as *MyoD* and genes encoding proteins for muscle structure, signalling, metabolism and transcription factors. Of the muscle CCGs identified, a large proportion was specific to each skeletal muscle. For example, antioxidant genes *Hmox1* and *Gsta3* were rhythmically expressed in the TA along with angiogenesis gene Angiopoietin-1 (*Angpt1)*, whereas enzymatic metalloproteinase family members, such as A disintegrin and metalloproteinase with thrombospondin motifs (*Adamts),* were rhythmically expressed in *Soleus* [83]; see Figure 2. Interestingly, mouse *Gastrocnemius* and human *Vastus lateralis* skeletal muscles shared 430 clock output genes, thereby suggesting conserved features of the skeletal muscle clock in both mouse and human muscle [70,84].

The circadian regulation of skeletal muscle has not only been demonstrated in murine models, but also in human studies. Human skeletal muscle plays an important metabolic role in the body, regulating proteostasis as well as glucose and lipid homeostasis. Desynchrony of human circadian rhythms, through irregular shift work patterns, leads to increased development of metabolic disorders such as obesity and type 2 diabetes [85]. Irregular shift work also increases the risk of skeletal muscle atrophy [86], whilst sleep disorders developed as a result of circadian desynchrony increase the degradation rate of skeletal muscle proteins [87]. Participants on a 14-day calorie restriction diet. who had undergone restricted sleep, lost 60% more muscle mass than those having regular sleep hours [88]. Furthermore, circadian desynchrony caused by insufficient sleep perturbed peripheral tissue clocks and led to impaired metabolism of skeletal muscle and liver as well as a whole-body imbalance in energy homeostasis. During such circadian desynchrony, hormesis is also affected, shifting the body to a catabolic state, thereby increasing the breakdown of skeletal muscle proteins and reducing the rate of protein synthesis [89].

Furthermore, in a study by van Moorsel and colleagues, a diurnal rhythm in skeletal muscle oxidative capacity was demonstrated in lean and healthy male participants [90]. Mitochondrial oxidative capacity peaked in the late evening (~23 p.m.) and troughed in the early afternoon (~13 p.m.). Interestingly, this aligned with the peak of resting energy expenditure (~23 p.m.-midnight) and coincided with the peak levels of core clock protein BMAL1 (~23 p.m.) and the trough of negative loop protein PER2, indicating a robust human skeletal muscle clock. In a follow-up study involving overweight male participants, mitochondrial respiration rates were lower when compared to the leaner participants, and the rhythmicity seen in the mitochondrial oxidative capacity was completely lost [91]. This suggests that restoration of the human muscle skeletal muscle clock may be an important step to improve metabolic health in those who suffer from metabolic disorders.

## 6. Circadian Regulation of Structural, Metabolic and Endocrine Functions of Skeletal Muscle with Ageing

Many studies have linked the disrupted genetic molecular clock to impaired skeletal muscle function, particularly with ageing. In 2006, Kondratov et al. demonstrated that a whole body knock-out of *Bmal1* induced early onset of sarcopenia, loss of skeletal muscle mass and strength with age, in addition to the loss of activity/rest rhythms and a reduction in locomotor activity [92]. *Bmal1* knock-out (KO) and *ClockΔ19* mice (with an ablation of exon 19 in the *Clock* gene) both exhibit ~30% reductions in normalised maximal skeletal muscle force, which is reciprocated at the single-fibre level, with changes in the myofilament architecture impairing contractile ability [66]. When a stabilising loop gene *Nr1d1* was knocked out in mice (encoding *Rev-erbα*), this resulted in a similar early ageing phenotype found by Andrews et al., (2010), with disrupted myofibre structure and an impaired ability to exercise [93]. Furthermore, using genetic approaches targeting *Bmal1* in the Duchenne muscular dystrophy (DMD^mdx^) mouse model, it was shown that a loss of *Bmal1* significantly accelerated dystrophic disease progression due to impaired myogenic progenitor proliferation [94]. In contrast, a loss-of-function of circadian repressor *Rev-erbα* in vivo augmented satellite cell proliferative expansion and regenerative progression during regeneration [95] through modulating Wnt/Notch signalling, and pharmacological inhibition of Rev-erbα (which stimulates *Bmal1* expression) ameliorates the severity of muscular dystrophy in mdx mice [96,97]. Similarly to *Rev-erbα* loss, satellite cell-specific knock-out of *Cry2* enhanced muscle regeneration by stimulating the proliferation of myoblasts and enhancing their differentiation potential [98]. Pre-clinical mouse models of DMD and muscle biopsies from DMD patients show deregulation of *Bmal1*, *Cry1* and *Cry2* in dystrophic muscles [99], which may result from disrupted dystrophin- RhoA-actin-SRF (serum response factor) signalling required for the molecular clock resetting [100]. Furthermore, deep RNA profiling of another muscular dystrophy caused by mutations in collagen VI A1-3 genes, using both the *Col6a1* knockout mouse model and patients with Collagen VI pathology [101], discovered alterations of the molecular clock genes, and specifically of the circadian gene CLOCK, as a key pathophysiological signature. These findings linking the altered molecular clock to muscular dystrophies could provide a rationale for exploring clock-augmenting interventions in conjunction with current or in-development therapies to ameliorate the dystrophic muscle phenotype by reinforcing sarcolemma integrity [102].

New research by Riley and colleagues (2022) has identified a mechanistic link between the molecular clock and the organisation of skeletal muscle sarcomere filaments. Using an inducible skeletal muscle-specific *Bmal1* KO, the group determined that the loss of *Bmal1* led to heterogeneity in sarcomere length due to altered titin isoform expression, caused by impaired transcriptional regulation of RNA-binding motif protein 20 (*Rbm20*), a splicing regulator of titin [72]. Titin provides passive stiffness to the sarcomeres and prevents overstretching during contraction, and dysfunction in titin has been linked to a range of skeletal muscle diseases including Late-onset autosomal dominant tibial muscular dystrophy, Limb–girdle muscular dystrophy type 2J and Adult-onset recessive proximal muscular dystrophy [103].

Further to impairments in skeletal muscle structure and contractility, a muscle-specific knock-out of *Bmal1* resulted in impaired insulin-dependent glucose uptake and reduced glucose oxidation in skeletal muscle as well as impaired systemic glucose homeostasis [76,104]. In line with skeletal muscle’s role in endocrine functions, it was found that basal secretion of cytokines such as IL-6, IL-8 and monocyte chemoattractant protein 1 (MCP-1) is under cell-autonomous circadian regulation in synchronised primary human skeletal myotubes, which was abolished when the molecular clock was disrupted through the knock-down of *Clock* [105] (see Figure 2).

Moreover, circadian regulation of skeletal muscle has revealed key roles in maintaining systemic health. Skeletal muscle plays a critical role in promoting systemic synthesis and accumulation of lipids in fat storage tissues such as adipose tissue and intestine through the cytokine unpaired 2 (*Upd2*), which maintains daily rhythms in circulating lipids in Drosophila [106]. More recent work has shown that scheduled exercise training led to marked modification of skeletal muscle clock gene expression and myokine production, in a manner that is dependent on the schedule of exercise training, which also impacted tumour progression [107]. Interestingly, muscle-specific *Bmal1* KO mice demonstrated a blunted transcriptional response to acute exercise, with gene expression signatures of inflammation across many tissues, suggesting that the skeletal muscle clock regulates systemic exercise effects [108]. Furthermore, rescuing *Bmal1* expression specifically in the skeletal muscle of *Bmal1* KO mice led to extended lifespan with improvements in skeletal muscle strength (but not muscle mass, fibre type or size), glucose tolerance, metabolic flexibility and reduction in inflammatory signatures across several peripheral tissues, including liver, lung and white adipose fat [109]. Moreover, exercise timing influenced the intra-tissue and inter-tissue metabolic signatures, revealing time- and tissue-dependent exerkines in liver and skeletal muscle, such as 2-hydroxybutyrate [110]. Therefore, disruption of the core clock genes in skeletal muscle leads to impaired structural, metabolic and endocrine functions of the skeletal muscle at the intrinsic cellular and systemic levels; see Figure 3. Given that daily voluntary exercise [111] and ageing [112] significantly remodel the murine muscle circadian transcriptomes, future work is warranted to decipher circadian muscle-secreted factors induced by exercise or ageing and their role in inter-tissue communication and systemic homeostasis.

## 7. Redox Homeostasis, NRF2 and Circadian Rhythms: A Bi-Directional Relationship

Cells and tissues maintain redox homeostasis and prevent RO/NS accumulation and associated oxidative damage through the activation of powerful antioxidant stress defence systems which sense variations in redox potential and trigger the required homeostatic processes to return to a steady state [113]. At low concentrations, RO/NS is considered to be a ‘eustress’ which is a physiologically beneficial oxidative stress [113]. Under these low oxidative stress conditions, RO/NS act as secondary messenger molecules and are known to activate several intracellular signalling pathways. RO/NS can enable adaptation to changes in environmental and internal redox environments via hydrogen peroxide signalling [114]. As skeletal muscle is a highly contractile and metabolic tissue, it requires large numbers of mitochondria to produce ATP. Due to aerobic respiration, skeletal muscle fibres are a major source of superoxide and nitric oxide, which can be converted into RO/NS [115]. When RO/NS levels rise above a physiological threshold, the oxidative stress ensues. Oxidative stress is thus a result of an imbalance between the production and the elimination of RO/NS, and represent a major risk factor for the onset and/or progression of vast array of age-related diseases including musculoskeletal conditions, cancer, cardiovascular and respiratory diseases, metabolic disorders (e.g., type 2 diabetes) and neurodegenerative conditions [116].

It is well established that there is coupling between the circadian clocks and redox systems, where the molecular clock directly regulates the levels of redox metabolites and antioxidant enzymes [117]. Self-sustained rhythms in the cellular redox state [92,118,119,120] as well as ROS-responsive antioxidant genes [121,122] have been observed in the brain and/or peripheral tissues. Moreover, diurnal rhythms in organismal survival to ROS-generating agents have been reported [123]. One of these antioxidant enzymes, Peroxiredoxin 1, is an evolutionarily conserved antioxidant protein that exhibits circadian redox oscillations in mouse tissues and in human red blood cells lacking nuclei [119,124], through a non-transcriptional/translational feedback loop.

Furthermore, there is a distinct interaction between the circadian clock and redox homeostasis via the NRF2-mediated redox signalling. The traditional paradigm of NRF2 regulation has mainly focused on oxidative stress-induced post-translational mechanisms, including regulation by its negative regulators (KEAP1, BACH1 and β-TrCP), positive regulators (DJ1, p62 and p21), or protein modifications [125,126,127,128,129]. However, the circadian paradigm of NRF2 regulation suggests that the endogenous levels of *Nrf2* are much more dynamic than previously thought. Indeed, *Nrf2* and its target genes have been identified as clock-controlled genes under the regulation of the core molecular clock in several peripheral tissues such as liver, lung, pancreas, macrophages and the brain, leading to rhythmic balance of redox homeostasis [130,131,132,133,134]. In this manner, the regulation of NRF2 nuclear activity appears to be restricted to a particular circadian window, gated by both the clock-controlled mRNA synthesis and the KEAP1-based protein degradation. 

In mouse liver, diurnal expression of mRNAs of *Nrf2* and *Nqo1* reached their highest expression in early evening (~6 p.m.), whilst troughing in early morning (~2–6 a.m.). Interestingly, the expression of NRF2 target genes was higher in females than males, especially for *Nqo1* [132]. Similarly, in mouse pancreas, *Nrf2* peaked in early evening (ZT14), and this regulation was abolished in *Bmal*1 KO-specific pancreatic islets (Zeitgeber ZT0= lights on, ZT12= lights off). In contrast, in mouse peritoneal cells, both *Bmal1* and *Nrf2* troughed during mid-afternoon (ZT8) and peaked in the early morning (ZT20), whilst a rapid induction of *Nrf2* was seen upon stimulation by BMAL1 [131]. Similarly to macrophages, in the mouse lung, *Nrf2* and its target genes *Gclm* and *Hmox1* peaked in the early morning (ZT0-3), whilst *Gsta3* peaked in the early evening (ZT15), suggesting that both transcriptional and post-transcriptional mechanisms may regulate tissue-specific antioxidant responses [130]). Further work in the mouse lung and fibroblasts demonstrated that *Nrf2* mRNA as well as its protein levels and nuclear activity are not only targets of the molecular clock under light/dark conditions, but are also regulated endogenously under constant darkness. Moreover, it was found that NRF2 itself rhythmically binds to promoters of antioxidant genes involved in glutathione synthesis (e.g., *Gclm/Gclc*) and utilisation (e.g., *Gsta3/4*), and that cyclical NRF2 levels were responsible for their rhythmicity [130]. This contrasts with an antioxidant gene *Prdx6*, which is cooperatively regulated by both BMAL1 and NRF2 in human lens epithelial cells [135].

Redox environment can also exert feedback on the molecular clock activity. The DNA binding activity of the CLOCK:BMAL1 heterodimer is dependent on the cellular redox status determined by the NADH/NAD^+^ and NADPH/NADP ratios, whereby binding is enhanced under reducing conditions [136]. NRF2 can also modify circadian clock gene expression and rhythmicity in mouse fibroblasts, hepatocytes and liver, indicating that NRF2 is involved in fine-tuning the timekeeping mechanism through a circadian clock-NRF2 bi-directional loop [137], although the precise mechanisms of this regulation have not been studied and require further studies in other peripheral tissues such as skeletal muscle.

## 8. Circadian Regulation of Mitochondrial Structure, Function and Dynamics

Mitochondria are vital for the production of energy in the form ATP through a process of oxidative phosphorylation, which is required for the functioning of many biological processes, including contraction of skeletal muscle [138]. The first stage of oxidative phosphorylation involves the oxidation of Nicotinamide Adenine Dinucleotide (reduced) (NADH) and Flavin Adenine Dinucleotide (reduced) (FADH2) via a series of electron transport chain protein complexes embedded in the inner-mitochondrial membrane. This produces an electrochemical gradient, with a higher concentration of H+ protons outside the mitochondrial membrane. The second stage involves the movement of the protons through the protein ATP synthase, inducing the binding of ADP (Adenosine Diphosphate) and phosphate (Pi), and the formation of ATP [139].

A comprehensive circadian proteomics study of mitochondria isolated from mouse liver identified that ~38% of mitochondrial proteins are rhythmic and oscillate through the day, peaking in protein abundance from ZT0-4 [140]. Both post-transcriptional and post-translational circadian mechanisms have been implicated in mitochondrial function and dynamics [141]. Circadian regulation of mitochondrial oxidative phosphorylation is achieved through several pathways: deacetylation of mitochondrial proteins by SIRT3, a NAD-dependent mitochondrial deacetylase [142] and via AMPK, a redox-sensitive energy sensor [143]. A global analysis of the liver acetylome in mice revealed CLOCK-dependent acetylation of enzymes within the Krebs cycle, glutathione metabolism and mitochondrial proteins [144], which are altered in *Bmal1* KO mice [142,145,146].

Furthermore, mitochondrial morphology and distribution are in constant flux, undergoing cycles of fusion (joining of mitochondria) and fission (separation of mitochondria), with fused mitochondria being more metabolically active as they have higher capacity for respiration [147]. Fission of mitochondria is dependent upon dynamin-related protein-1 (DRP1), which is regulated through transcriptional and post-transcriptional mechanisms (phosphorylation) in a circadian manner as demonstrated in human skin fibroblasts as well as the mouse brain and liver, whilst mitochondrial fusion is regulated through circadian post-transcriptional regulation of fusion proteins MFN1/2 and OPA1 [148]; see Figure 2.

Mitochondrial membrane potential also follows a circadian pattern as demonstrated in murine macrophages, gradually increasing 4 h after serum shock and peaking at 12 h to 16 h before gradually depolarising for the rest of the circadian cycle [149]. The circadian clock also regulates the rhythmic synthesis of NAD+, thereby producing oscillations in the mitochondrial capacity for energy production [150].

The balance between mitochondrial biogenesis and mitophagy determines the mitochondrial content of the cell. PGC-1α/β are key regulators of mitochondrial biogenesis whilst PTEN-induced kinase 1 (PINK1) and Parkin RBR E3 ubiquitin ligase (PARKIN) are essential in mitophagy [151]. PGC-1α/β are rhythmically expressed in the liver and skeletal muscle in mice, with functioning PGC-1α being required for cell-autonomous clock function [152]. *Pink1* is a direct target of BMAL1, and in human skeletal muscle, PINK1 protein levels peak in the middle of the active phase [149]; see Figure 2. In synchronised human fibroblasts, transcription of *Park2* (coding for PARKIN) and *Park6* (coding for PINK1) displayed oscillations in expression, with ~24 h periods [153]. The oxygen consumption rate, a readout of mitochondrial respiration, exhibits circadian oscillation, which is lost in the liver mitochondria of *Per1/2* KO and *Bmal1* KO mice [140,142,148]. Additionally, PER1 and PER2 regulate the key rate-limiting mitochondrial enzymes, such as carnitine palmitoyl-transferase 1 (CPT1) in mouse liver, which are required for the processing of nutrients in a time-of-day manner [140]. Circadian clock components also affect lifespan determination in *Drosophila* by altering cellular respiration via mitochondrial uncoupling [154], suggesting that the mitochondrial metabolism and circadian rhythms are coupled at a systemic level [155].

*Bmal1-/-* and *Clock^Δ19^* mice show a ~40% reduction in mitochondrial volume in skeletal muscles, and of the mitochondria present, most were abnormal in shape, impairing their ability to undertake respiration and produce ATP. These mice also show altered expression levels of *Pgc1α/β* [66]. Exercise and melatonin were able to restore myogenesis and altered mitochondrial dynamics associated with sarcopenia in *Bmal1* KO mice [156]. *Nr1d1-/-* mice show a similar reduction in mitochondrial volume in skeletal muscles [93]. Deficiency of *Rev-erbα* in mice induced increased mitophagy through a lack of inhibition of *Park2* and Unc-51 like autophagy activating kinase 1 (*Ulk1*), shifting the mitochondrial dynamics, reducing energy capacity and resulting in a decline of total mitochondrial content [93]. Mice with a double knock-out of *Cry1*/*2* are capable of running further and faster than WT littermates, enhancing exercise capacity due to increased peroxisome proliferator-activated receptor δ (PPARδ) activity and fatty acid oxidation [157]. Furthermore, muscle-specific *Per2* deletion in mice led to a lower glucose tolerance during the day compared with WT littermates but no difference was seen during night-time. Night-time activity levels were also significantly reduced in *Per2* KO mice but this did not affect exercise capacity [158]. Circadian clock dysregulation is observed in primary myocytes and skeletal muscle biopsies in individuals with type 2 diabetes and is linked to altered rhythmic mitochondrial respiration and rhythmic transcripts of the mitochondrial inner membrane [159]. However, the circadian mechanisms of mitochondrial structure, function and dynamics in skeletal muscle have not been studied in detail, and whilst some of the circadian insights from other peripheral tissues are likely to operate in skeletal muscle, further studies are warranted, especially when it comes to responses to exercise and ageing.

## 9. Extracellular Matrix Homeostasis Regulation by the Molecular Clock

The ECM plays a major role in determining the stiffness of the skeletal muscle microenvironment during rest and in mechanical responses to exercise. Excessive accumulation of ECM in the skeletal muscle with ageing leads to increased stiffness, which inhibits the myogenic differentiation capacity of muscle-resident stem cells. The ECM components required for maintaining the SC niche are altered in the skeletal muscles of aged mice. Old muscles show increased collagen content by ~35–40% of the extracellular space with a higher proportion of type-I collagen to type-III collagen as well as increased proportions of collagens type IV and VI alongside increased levels of the non-enzymatic crosslinking of collagen fibres such as glycosylation. This leads to changes in the elastic modulus of the ECM from ~12 to ~ 418 kPa in older skeletal muscles, demonstrating much stiffer microenvironment, that affects both stem cell activity and generation of skeletal muscle force [10].

Skeletal muscle stem cells, known as satellite cells, reside in the niche within the ECM, and they are vital for the regeneration of skeletal muscle, maintaining strong oscillations of the circadian clock machinery [160]. Circadian time-series proteomics revealed that many ECM proteins show daily oscillations in their abundance including collagens and various components of the matrisome in the mouse femoral head cartilage [161]. Research by Chang and colleagues identified that key regulators of collagen type I secretion, including transport and Golgi organisation protein 1 (TANG01) and SEC61, are rhythmic in their expression, resulting in nocturnal procollagen-1 synthesis with daytime collagen type I fibril assembly, thus affecting oscillations of a pool of newly synthesised collagen type I. Arrhythmic *Clock*^∆19^ and *Bmal1* KO mice both accumulate *Col1α1* mRNA and have a disorganised and structurally and mechanically abnormal collagen matrix [162]. Matrix metalloproteinase-14 (*Mmp14*), a key protease involved in the remodelling of ECM through the degradation of macromolecules such as type I collagen, is also rhythmically expressed at both mRNA and protein levels. A loss of *Mmp14* leads to arrhythmic tendon fibroblasts and severe fibrosis of the ECM [163]. Dysregulation of various ECM proteins and tissue fibrosis have been seen in a wide range of peripheral tissues in both *Bmal1* KO and *Clock^∆19^* mice including: lungs [130,164,165,166,167], heart [168,169,170,171,172], skeletal muscle [75], kidney [173,174,175], liver [176,177,178,179,180], pancreas [181] and adipose tissue [182,183]; see Figure 3. Inducible skeletal muscle-specific *Bmal1* deletion also resulted in increased muscle fibrosis, together with increased bone calcification and alterations in joint knee collagens IV and V [75]. Moreover, ageing of several peripheral tissues leads to diminished circadian regulation, with changes in the ECM content and associated fibrosis [184,185,186,187,188]. Therefore, the circadian clock regulates the function of connective tissue components within the microenvironment such as residing tissue-specific stem cells as well as the abundance and activity of collagens and ECM proteins, although the circadian regulation of ECM in skeletal muscle, and how it changes with ageing specifically, has not been studied and warrants further research.

## 10. Chronotyping: Personalisation of Skeletal Muscle Performance

A ‘chronotype’ refers to the phase of an individual’s inner biological clock. It differs between individuals such that the behavioural preference for one individual might be to be most active in the morning, whilst for another it may be in the evening [189]. A chronotype is influenced by physiological, genetic and behavioural factors and can be categorised into three groups, with morning types or ‘early birds’ on the one end, evening types or ‘night owls’ on the other, and an intermediate type in between. Morning types are typically phase advanced, being most active in the morning and early hours of the day, whilst the evening types are most active and alert in the later hours of the day. The intermediate types have the most flexibility, having a greater capacity to adapt to a required delay or advance depending on lifestyle requirements. A study by Facer-Childs and Brandstaetter (2015) involved early-, intermediate- and late- types undertaking a progressive aerobic endurance test at 7 a.m., 10 a.m., 1 p.m. and 10 p.m. The early types performed best at ~12.10 p.m., the intermediates at ~15.50 p.m. and the late types at ~19.40 p.m., and there was performance variation between the groups over the day; ~7–10% for the early and intermediates, but up to 26% for the late types [190].

A systematic review by van der Merwe et al. (2022) investigated how different chronotypes affect body composition and biomarker outcomes. They found that the evening chronotypes had increased body mass index and increased weight gain over time when compared to early chronotypes [191]. Moreover, individuals with late chronotype may have better cognitive and motor performances in the evening compared to earlier times of day [192]. Evening type swimmers, for example, showed a reduced performance in the morning compared to the evening, with both morning and evening types displaying higher stress levels when training at times opposite to their chronotypes [193]. Similar observations were seen in morning chronotype cyclists performing in the evening who reported higher exertion rates [194].

Chronotypes can be entrained to a degree; morning exercise can help to advance a late chronotype's phase to improve their morning performance [195]. Indeed, Facer-Childs and colleagues investigated if a combination of timed light exposure, sleep, meals, caffeine and exercise could shift the phase of ‘night owls’ in a real-world setting. Using a carefully formulated regimen, the researchers were able to achieve a phase advance of ~2 h in the participants, who also reported reduced morning sleepiness and decreased rates of depression and stress alongside improved cognitive (reaction times) and physical (grip strength) performance [196]. Interestingly, whilst late chronotypes may benefit from phase advances induced by exercise in the morning or evening, evening exercise may exacerbate circadian misalignment in early chronotypes [197]. Additionally, for athletes participating in team sports, there is evidence that chronotype distribution within teams can influence team performance [190]. Thus, whilst chronotypes can influence many physiological processes, new studies are required to understand how best to optimise this knowledge, especially if chronotypes are to be incorporated into personalised exercise regimens.

## 11. Time-Scheduled Exercise as a Lifestyle Intervention for Skeletal Muscle Loss with Ageing?

Currently, exercise is one of the key promising treatments for skeletal muscle loss with ageing as it can target both the metabolic and functional deficits of skeletal muscle [198].

Acute and chronic exercise increase mitochondrial function in skeletal muscle of rats [199] and humans [200]. Whilst contracting skeletal muscles during exercise produce more RO/NS, it has been determined that RO/NS itself are required to trigger and mediate the adaptive responses to exercise [201]. However, a single acute bout of exercise can generate excessive amounts of RO/NS which can lead to oxidative damage within the skeletal muscle [201]. Conversely, regular exercise can induce adaptations to resist oxidative damage through the activity of antioxidant pathways [201].

Aerobic exercise training in young adults and older individuals was capable of increasing *Nrf2* signalling in both groups as well as of lowering the basal level of *Nrf2* in the older group [202]. Moreover, in humans, baseline NRF2 expression in skeletal muscle correlates with maximal oxygen uptake and high-intensity exercise performance [203]. A systematic review investigating exercise-inducing adaptations identified that exercise training also promoted significant increase in mRNA and protein expression of various collagens, including types I, III, IV and VI collagens, in young adults and older participants [204]. Older participants experienced a higher fold change in ECM content compared to young adults during high intensity interval training (HIIT), including changes in collagens IV and XIV, elastin and periostin [205].

Optimal redox responses to exercise require functional NRF2 signalling. Mice with a *Nrf2* loss in muscles perform significantly worse in treadmill exercises and show impaired oxidative/glycolytic muscle contractility, whilst loss of *Keap1*, a repressor of *Nrf2*, enhanced exercise capacity and muscle contractility [206]. In line with this, treatment of rats with *Nrf2* activator, sulforaphane, enhanced running capacity and diminished muscle fatigue by upregulating *Nrf2* signalling and increasing downstream antioxidant gene expression [207]. Conversely, in a model of chronic *Nrf2* activation through systemic loss of *Nrf2* regulator *Keap1*, exercise endurance capacity in mice was increased [208].

Exercise is an external cue capable of synchronising clock gene expression in skeletal muscle. Recent work has demonstrated that daily variations in the expression of human core clock genes can predict fluctuations in exercise performance across the day [209]. Moreover, the timing of aerobic exercise impacts the levels of BMAL1 and NRF2 protein expression. Mice that exercised at Zeitgeber time 12 (ZT12, ZT0 = lights on, ZT12 = lights off) exhibited higher total antioxidant capacity and lower RO/NS in skeletal muscles than mice that exercised at ZT24. Antioxidant proteins NQO1 and HMOX-1 were both significantly upregulated at ZT12 compared to ZT24, suggesting a more adaptable skeletal muscle clock and timed antioxidant response to exercise [210].

Daily voluntary running also dramatically remodels the mouse skeletal muscle circadian transcriptome, with skeletal muscle and liver glycogen exhibiting robust daily rhythms [111]. These authors determined that daily voluntary wheel running of male mice resulted in an increased maximum speed at ZT21, at the end of the active phase, but not at ZT13, at the start of the active phase. The active mice also gained less weight than their age- and sex-matched sedentary counterparts. Interestingly, this time-of-day difference was seen in male mice but was not observed in age-matched females, though females were overall more active and had greater maximum sprint speed than males [111]. When investigating metabolite levels at ZT13 and ZT21, there were stable levels of glucose and β-hydroxybutyrate (major energy sources particularly when the body is low on carbohydrates) at ZT21 along with increased blood lactate, but a significant reduction in glucose at ZT13. The authors suggested that maximum speed at ZT21 is associated with increased selective use of carbohydrates for energy production [111]. This is consistent with a human study by Iwayama et al. (2021) where participants showed increased respiratory exchange ratio (measure of utilisation of carbohydrates and fats during energy production) when undertaking a single bout of 60 minute cardiovascular exercise in the afternoon (4 p.m.) when compared to the morning (7 a.m.) [211]. Interestingly, afternoon HIIT was more efficacious than morning HIIT at improving blood glucose in men with type 2 diabetes [212], and it also led to increased skeletal muscle lipids and mitochondrial content, the clinical implications of which warrant further research [213]. In contrast, exercise responses during the early active phase (ZT12) are characterised by HIF1α-dependent activation of glycolytic pathways in the mouse skeletal muscle, resulting in carbohydrate exhaustion, usage of alternative energy sources and adaptation of systemic energy expenditure [214].

Daily voluntary running also dramatically remodels the mouse skeletal muscle circadian transcriptome, with skeletal muscle and liver glycogen exhibiting robust daily rhythms [111]. These authors determined that daily voluntary wheel running of male mice resulted in an increased maximum speed at ZT21, at the end of the active phase, but not at ZT13, at the start of the active phase. The active mice also gained less weight than their age- and sex-matched sedentary counterparts. Interestingly, this time-of-day difference was seen in male mice but was not observed in age-matched females, though females were overall more active and had greater maximum sprint speed than males [111]. When investigating metabolite levels at ZT13 and ZT21, there were stable levels of glucose and β-hydroxybutyrate (major energy sources particularly when the body is low on carbohydrates) at ZT21 along with increased blood lactate, but a significant reduction in glucose at ZT13. The authors suggested that maximum speed at ZT21 is associated with increased selective use of carbohydrates for energy production [111]. This is consistent with a human study by Iwayama et al. (2021) where participants showed increased respiratory exchange ratio (measure of utilisation of carbohydrates and fats during energy production) when undertaking a single bout of 60 minute cardiovascular exercise in the afternoon (4 p.m.) when compared to the morning (7 a.m.) [211]. Interestingly, afternoon HIIT was more efficacious than morning HIIT at improving blood glucose in men with type 2 diabetes [212], and it also led to increased skeletal muscle lipids and mitochondrial content, the clinical implications of which warrant further research [213]. In contrast, exercise responses during the early active phase (ZT12) are characterised by HIF1α-dependent activation of glycolytic pathways in the mouse skeletal muscle, resulting in carbohydrate exhaustion, usage of alternative energy sources and adaptation of systemic energy expenditure [214].

Daily scheduled voluntary exercise can also affect SCN’s molecular and neuronal activities, thus promoting cellular clock synchrony and robust ~24 h rhythms in behaviour [215]. In mice lacking neuropeptide vasoactive intestinal polypeptide (VIP) in the SCN, which leads to blunted and temporally disorganised molecular clocks, daily voluntary exercise promoted SCN clock cell synchrony and robustness of ~24 h rhythms in behaviour [215].

Furthermore, specific clock proteins can modify exercise capacity in a time-dependent manner, likely through differences in food consumption and liver glycogen stores, with BMAL1 impairing and PERIODs enhancing exercise capacity in a time-dependent manner [216]. Moreover, muscle-specific *Bmal1* knock-out mice present with diminished transcriptional responses to acute exercise as demonstrated through limited upregulation of exercise-responsive transcription factors Neuron-derived orphan receptor 1 (*Nr4 a3*) and *Pgc1α* [108]. Research has demonstrated that contraction of skeletal muscle through exercise leads to increases in cAMP levels, which activates cAMP response element binding protein (CREB) that, in turn, activates circadian PERIOD1/2 proteins, whilst the generated RO/NS activate a NAD-dependent protein deacetylase Sirtuin-1 (SIRT1) and PGC-1α to bind to circadian transcription factor RORα/γ, both pathways improving exercise performance [67]. Moreover, synchronisation of aerobic exercise with the circadian rhythm of NAD^+^-SIRT1 boosts MFN2-mediated mitochondrial fusion by activating the BMAL1-PER2-SIRT1-PPARα axis in the skeletal muscle of diabetic mice, leading to more effective glycaemic control and improved insulin resistance [217].

It is important to note that different forms of exercise rely on enhancing distinct physiological processes and functions of skeletal muscle. Resistance exercise (e.g., weightlifting) promotes anabolic responses and muscle hypertrophy by increasing muscle mass through the activation of mTOR pathway which regulates the balance between protein synthesis and degradation [218]. Endurance exercise (e.g., marathon running) improves skeletal muscle function without substantial hypertrophy by improving maximum volume of oxygen (VO_2_ max) and stimulating mitochondrial biogenesis to optimise aerobic metabolism efficiency [219]. Therefore, different exercise regimens may be more optimal to undertake at specific times-of-day. For example, human skeletal muscle torque, strength and power is increased during the late afternoon (between 16 and 18 p.m.) compared to the morning [190]. In a study of Olympic swimmers, athletes produced more competitive results when competing in the afternoon races, particularly ~17 p.m. [220]. Semi-professional female volleyball players exhibited increased standing spike velocity, flexibility, dynamic balance and agility at 19 p.m. in comparison to 9 a.m. [221]. Male professional tennis players jumped higher and were faster during the afternoon [222], and male Olympic weight-lifters [223] and male professional rugby players [224] both demonstrated a better performance and a higher output during the afternoon. Conversely, professional marathon runners produced improved personal record times at 6.30 a.m. in the morning [225]. For a comprehensive review on the influence of circadian rhythms on different sports performances, please refer to [226].

Many studies have demonstrated regular exercise as a strategy to improve sarcopenia and physical functions in the elderly. Landi et al. (2014) provided a comprehensive review of different exercise regimens used as treatments for sarcopenia and concluded that physical exercise increases aerobic capacity, skeletal muscle strength and endurance in older individuals [227]. Indeed, a search for ‘sarcopenia’ and ‘exercise’ on the Clinical Trials website (www.clinicaltrials.gov) produced 424 trials. A total of 409 studies were targeting older adults (65+ years) and 392 classed ‘exercise’ as an intervention. There was a variety of exercise interventions being investigated, including high/low loads, pilates, resistance, high intensity and home-based physical activities. Exercise is therefore a very promising intervention for skeletal muscle loss with ageing as it can improve functional and metabolic aspects of skeletal muscle and ameliorate the deterioration of energy metabolism with age. Resistance training is potentially of most benefit to the elderly as it increases skeletal muscle mass and strength to improve quality of life; however, the elderly typically have earlier chronotypes. Therefore, it can be postulated that the afternoon resistance exercise may be better adapted to earlier times in the day for the elderly populations. However, how time-of-day exercise responses change with ageing and age-related chronotype shifts is currently unknown and further research is warranted to maximise the therapeutic potential of chrono-based exercise interventions.

## 12. Sex-Tailored Exercise Interventions for Sarcopenia and the Role of Circadian Hormone Melatonin

Whilst the above studies demonstrated that circadian rhythms are paramount in optimising athletic performance, it is worth noting that most of these studies were undertaken by male participants. Inclusion of women in research studies was not promoted by the National Institutes of Health (NIH) in the USA until 1986, therefore prior to this date only male organisms and isolated cells were used in research. Pre-1993, the US Food and Drug Administration banned women of childbearing age to be included in clinical trials; thus, men were the main subjects of research. Indeed, historical bias, costs, concerns for decreasing fertility and arguments of hormonal fluctuations impacting findings are some of the reasons behind scarcity of studies on the female population [228]. This sex bias was also present during the animal testing stages of research. Between 1990 and 2009, 80% of all rodent studies used exclusively males. Some arguments against the use of female animal models were similar to human, mainly related to challenges in controlling hormonal fluctuations. However, there were additional reasons including female animals being required as breeders, making males surplus and more readily available for experiments [229]. In 2014, the NIH issued a mandate that all studies which were awarded grants were to include sex as a biological variable in cell lines, tissues and organisms, unless biological sex was not relevant, though this needed to be proved through strict logic and analysis [229].

Systematic reviews by Ose et al. and Paul et al. highlighted this sex imbalance in human studies [230, 231]. Paul and colleagues reviewed 669 studies from 2017 to 2021 across six top sports medicine journals and found that 70.7% of the studies focused on male-only athletes, whilst 8.8% were female-only, with males dominating in studies on sports such as football, rugby and basketball, and women more commonly studied in softball and volleyball studies [230]. Ose and colleagues investigated 1441 original and epidemiological research studies published in three major sports and exercise medicine journals between 2021 and 2023, totalling ~40.1 million participants. The mean proportion of female participants was 40.22%. However, only 103 (7.15%) of studies were female-only, and of them, a total of 66 (5.6%) included menstrual-status considerations in their study design [231]. Whilst women are 2:1 to 9:1 more likely than men to experience anterior cruciate ligament (ACL) injuries during sport activities, male athletes still predominate research population in this research field [232].

Thus, an important consideration when investigating the effects of ageing on skeletal muscle is the sex of the participants as there are many sex-specific differences in skeletal muscle maintenance, many of which stem from hormonal regulation at the onset of puberty. For example, males typically have more muscle mass than females, regardless of activity levels, and females have a greater proportion of type I muscle fibres whereases males have a larger proportion of type II fibres [233], possibly due to greater hypertrophy of type II fibres induced by testosterone [234]. Some metabolic properties of skeletal muscle also differ between males and females. Women use more lipids during exercise than men do, with higher levels of intramuscular triacylglycerol and fatty acid translocase proteins present in women than men [234]. Sex also influences skeletal muscle calcium (Ca^2+)^ regulation; Ca^2+^-ATPase activity was reduced with exercise in male participants but increased in women. This may be due to differences in proportions of fibre types, with type I fibres having lower maximum sarcoplasmic reticulum Ca^2+^-ATPase activity, suggesting that female skeletal muscle is more likely to resynthesise ATP from oxidative phosphorylation during exercise [235]. Moreover, myofibre size is also influenced by sex. Across all myofibre types, cross-sectional area is greater in males than females, and this difference appears during the transition from childhood to adulthood [236]. Recent research has shown that type II myofibres in the *Vastus lateralis* muscle of untrained adult males contained more satellite cells than those of their female counterparts [237]. This was supported by murine studies where the number of satellite cells in the *Extensor digitorum longus* muscle of male mice, predominantly composed on type II fibres, was greater than in age-matched females [238].

A key difference between male and female skeletal muscle structural/functional properties lies with the sex-specific hormones present in the body, and/or circulating hormones levels at very different concentrations. Both sex hormones regulate key physiological processes including postnatal muscle growth, promoting contractility and governing the hypertrophic response to resistance exercise [233]. Testosterone binds to nuclear androgen receptors, promoting development of type II over type I fibres, upregulating glucose clearance and glycogen synthesis and enhancing muscle mass and strength. The cellular mechanism for the activity of testosterone involves the upregulation of follistatin, a protein which inhibits the activity of myostatin, restricting skeletal muscle growth [233]. Testosterone also impacts skeletal muscle contractile function, with supplementation of the hormone increasing isometric knee extension peak torque in older male humans [239].

When oestrogen binds to the oestrogen-receptor complex, it promotes preferential development of type I over type II myofibres, mitochondrial dynamics and bioenergetics. Additionally, it maintains the pool of satellite cells, skeletal muscle contractility and protects against muscle damage and inflammation [233]. Whilst the action of testosterone in skeletal muscle is well described in the literature, the actions of oestrogen in the same context remain to be elucidated. Oestrogen has a positive effect on skeletal muscle regeneration after injury whilst decreasing exercise-mediated skeletal muscle injury through inhibition of inflammatory responses and increased growth of satellite cells [240]. Oestrogen is important in the regulation of mitochondrial function in skeletal muscle. Female mice with a muscle-specific knock-out of oestrogen receptor-α have reduced basal and stimulated oxygen consumption rates, excessive RO/NS production and mitochondrial morphological abnormalities including enlargement, elongation and hyper-fusion [241].

Melatonin is a highly conserved circadian ‘dark’ hormone synthesised in the pineal gland and most tissues and organs of the body, including skeletal muscle. Melatonin produced by the pineal gland oscillates in synchrony with the light/dark cycle and is controlled by the master clock in the SCN, though extrapineal melatonin does not follow a circadian rhythm [242]. Melatonin is a very versatile molecule, with pineal melatonin serving chronobiological functions and extrapineal melatonin acting as an antioxidant and anti-inflammatory molecule [242]. Both pineal and extrapineal melatonin decrease with age, the former leading to a decline in circadian control of peripheral tissues and the latter linked to mitochondrial dysfunction [242], suggesting that melatonin administration could be used as a potential therapy against sarcopenia.

Young male footballers treated with melatonin prior to intense exercise showed reduced levels of lipid peroxidation when compared to controls and also showed increased total antioxidant activity for up to 60 minutes post-exercise [243]. In rats treated with melatonin and subjected to exercise, melatonin led to enhanced exercise tolerance, increased expression of PGC-1α, decreased skeletal muscle triglyceride content and increased muscle glycogen levels 3 hours post-exercise [244]. Treatment of aged mice with melatonin improved function and structure of the skeletal muscles whilst reducing damage to mitochondria [245]. In muscle-specific *Bmal1* knock-out mice, which express hallmark features of sarcopenia (circadian and mitochondrial disruption and impaired skeletal muscle function), combined exercise and melatonin treatment reversed these effects independently of *Bmal1*. In addition, mitochondrial dynamics was improved and myogenesis was restored [156]. In male rugby players, core clock genes *Bmal1*, *Rorα*, *Cry1*, *Per1*, *Per2* and *Nr1d1* were upregulated compared with sedentary controls, and melatonin levels oscillated over the day in athletes, but remained constant in sedentary males [246]. Thus, a potential dual therapy of exercise and melatonin supplementation could be a therapeutic strategy to reduce the effects of sarcopenia in the elderly. Currently, there are 969 clinical trials being undertaken with melatonin as the intervention, many of which are for sleeping problems; of these, 15 are investigating melatonin's anti-ageing effects, though most are either for neurodegenerative disorders or osteoporosis. Only one study investigated melatonin in relation to sarcopenia (NCT03784495), and no studies investigated sarcopenia, exercise and melatonin. Thus, more preclinical and clinical research is warranted before dual melatonin/exercise therapy could be tested as a therapeutic treatment for sarcopenia.

A recent UK biobank study revealed that whilst there was a significant age-related decline in skeletal muscle strength in both males and females, loss of muscle mass was more pronounced in males, whilst females displayed substantially greater age-related decline in quality of skeletal muscle [247]. The cross-sectional area of the *Quadriceps* has been shown to decrease in both sexes, but more significantly in women [248]. Older adults have decreased intermyofibrillar mitochondrial size, however, the age effect was mostly seen in women. Additionally, the slowing of myosin cross-bridge kinetics is particularly observed in elderly women [249]. The review by Della Peruta et al. (2023) details the sex differences in skeletal muscle in adulthood and in relation to ageing [250].

Furthermore, elderly men and women both benefit from prolonged (six months, three times per week) resistance training, which leads to increases in leg lean mass and *Quadriceps* cross-sectional area [251]. There are currently 244 clinical trials registered on Clinical Trials website (clinicaltrials.gov) when searching the parameters such as ‘ageing’, ‘exercise’ and ‘skeletal muscle’. However, when ‘circadian’ is added to the search parameters, this number drops to three studies only. Of the three studies, only two, NCT06136013 and NCT07075133, specify the use of exercise in the morning or afternoon as the intervention. This demonstrates that whilst exercise is considered an important therapeutic intervention for the treatment of sarcopenia, there is a great need for further research into time-of-day exercise interventions in the elderly. Therefore, ageing studies using exercise as an intervention warrant inclusion of both circadian timing and sex of participants as important variables to be studied as circadian timing of exercise may affect the responses of different types of exercise whilst sex of participants may influence benefits from interventions targeting fibre size and quality, rather than quantity alone.

## 13. Summary

**Figure 4:**
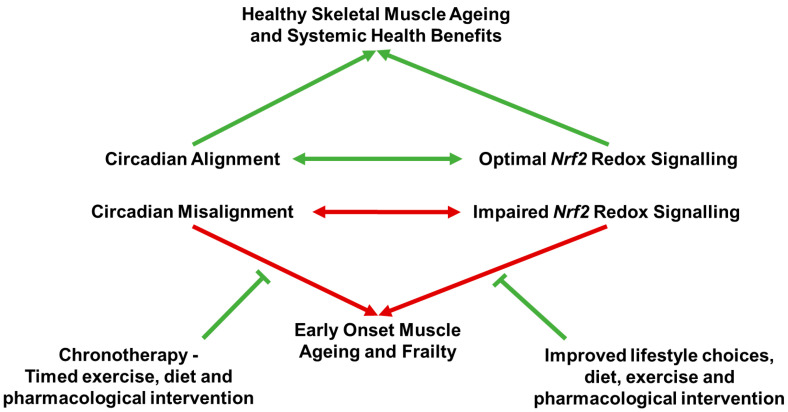
Schematic showing proposed bi-directional interactions between the circadian clock and *Nrf2*-based redox signalling to regulate the rate of skeletal muscle ageing and systemic health. Under appropriate circadian alignment, the *Nrf2* redox signalling can function optimally, which supports long-term maintenance of systemic health and healthier ageing of skeletal muscle. Circadian misalignment or impaired *Nrf2* redox signalling leads to pathophysiological consequences in the skeletal muscle, such as the accelerated development of sarcopenia and systemic loss of tissue resilience to stressors leading to frailty. Using chronotherapy-based approaches, including timed exercise, diet or pharmacological interventions, along with improved lifestyle choices, circadian alignment can be achieved alongside optimal redox homeostasis to promote healthy ageing of the skeletal muscle and systemic health benefits.

Both the circadian molecular clock and *Nrf2* redox signalling regulate skeletal muscle function, metabolism and mitochondrial activity, with growing evidence demonstrating the importance of these systems in age-related skeletal muscle decline and systemic loss of tissue resilience. Many tissues, including skeletal muscle, display both circadian dysfunction and redox imbalance during ageing [252,253,254,255], therefore future exercise regimens may maximise skeletal muscle functional and systemic health benefits by incorporating chronotherapy-based strategies to boost both the molecular clock robustness (i.e., circadian alignment) and *Nrf2*–mediated antioxidant system through timed exercise, diet or pharmacological interventions. Figure 4 depicts a proposed model of how boosting circadian alignment, through time-scheduled lifestyle approaches such as exercise, may delay the onset of and/or ameliorate skeletal muscle ageing and frailty when coupled with other lifestyle improvements such as diet and pharmacological supplementation (e.g., melatonin).

Recently, Zhang et al. identified that Achilles tendinopathy (AT) disrupts the *Achilles* tissue clock, reducing both *Bmal1* and *Nrf2*, which further exacerbated tissue inflammation. Enhancing *Bmal1* expression mitigated tendinopathy and increased AT relief [256]. Moreover, the timing of aerobic exercise modulates the levels of BMAL1 and NRF2 protein expression alongside the total antioxidant capacity and RO/NS levels. Furthermore, research has highlighted that AMPK signalling in adipocytes impacts exercise-induced beneficial effects on skeletal muscle function in a time-of-day manner by affecting skeletal muscle metabolites, thus implicating an important role for circadian inter-tissue cross-talk in exercise responses [257]. Despite this promising research, Palmese and colleagues (2025) report in a systematic review a relative lack of research undertaking circadian timing approaches in combating sarcopenia. They concluded that whilst available studies supported the role of the circadian timing system in sarcopenia, the research was limited [258]. Their suggestion is that researchers take advantage of published studies to optimise and refine experimental and clinical protocols, using clinical and preclinical models to address the wide knowledge gaps in the field.

Exercise remains the most promising intervention for sarcopenia, yet its effectiveness may vary depending on many external factors, including feeding/fasting cycles and the time of day exercise is performed. A total of ~20% of people do not improve their respiratory capacity when they exercise regularly; they are referred to as “non-respondents”. Additionally, ~7 to 15% of people experience adverse reactions when exercising on a regular basis [259], including increased systolic blood pressure, increased blood levels of triglycerides and insulin, and lower HDL cholesterol [260]. Despite this variability in exercise responses, few experimental studies and clinical trials account for circadian timing and personal chronotypes as important variables. Yet, appropriately timed exercise may improve health outcomes and reduce the frequency and/or duration of exercise needed, potentially lowering heterogeneity in exercise responses, especially given that circadian regulation is often phase-shifted in older adults.

Whilst outside the scope of this review, diet is an important factor to consider in future studies of skeletal muscle exercise, ageing and circadian biology. Lipid, glucose and amino acid metabolism are all regulated by the circadian clock and change with age; see reviews [261,262]. The timing and nutritional composition of food intake are important Zeitgebers for the circadian clocks in humans [262]. Meal frequency and distribution within a day are highly related to metabolic functions, and optimal time-restricted feeding has the potential to prevent several metabolic dysfunctions. Thus, eating at an inappropriate time (e.g., during the night) may have a desynchronizing effect on the circadian clocks and, in the long term, may result in adverse health outcomes (e.g., weight gain, obesity and poor metabolic function). The digestive, absorptive and metabolic capacities of nutrients also show the day–night variations in several peripheral tissues such as small intestine and liver. For example, the postprandial response of blood triacylglycerol to a specific diet and glucose tolerance exhibit clear time-of-day effects. Ingestion of carbohydrates increases secretion of insulin which, together with insulin-like growth factor 1 (IGF-1), can entrain cellular clocks by increasing the synthesis of PERIOD proteins alongside mechanistic target of rapamycin (mTOR) activation [263].

Diet and exercise are intrinsically linked. The nutritional composition of food intake influences the exercise responses. High protein diets enhance muscular performance and skeletal muscle mass in resistance-trained males, irrespective of protein intake immediately prior to exercise or 3 h post exercise [264]. Dietary protein is also required to repair damaged cells and tissues, synthesise hormones and support metabolic activities. Protein ingestion pre- or post-exercise has been shown to enhance recovery, immune function as well as growth and maintenance of lean body mass [265]. Beneficial synergistic effects of exercise and time-restricted feeding have been reported for mice fed a high-fat diet [266].

Older adults are at increased risk of malnutrition due to a ~25% decline in food intake with age, leading to energy supplies lower than the recommended values [267]. Insufficient protein intake is a common nutritional deficit seen in the elderly leading to lower skeletal muscle protein synthesis. It is currently suggested that the elderly ingest 1–1.3 g/kg/day of protein to sustain skeletal muscle mass and function [268]. For building muscle mass, overall protein intake should be 1.4–2 g/kg/day, and if this is not possible through food consumption, supplementation is recommended [269]. Thus, optimising nutritional intake could be a vital piece of puzzle in designing improved exercise regimens, especially for the elderly.

To maximise the benefits of exercise-based lifestyle interventions for musculoskeletal health, future studies should consider circadian timing of specific exercise regimens and diet intake/composition alongside sex, age and chronotype, as these factors interact in complex ways to influence health outcomes and are prone to misalignments between the circadian system and environmental cues (e.g., reduced physical activity, consuming energy-dense nutriments, irregular eating, disruptions in sleep patterns, shift work, light pollution). For example, resistance training appears optimal in the afternoon for skeletal muscle growth but may be better tailored to earlier times of day for individuals with an early chronotype. Recognising the complex interplay between physiological, chronobiological and nutritional factors will be essential for developing more effective personalised lifestyle and therapeutic strategies to prevent and/or treat age-related skeletal muscle function decline and its associated systemic health effects.
